# Standard E TB-Feron ELISA and Standard F TB-Feron FIA positivity rates and agreement with QuantiFERON-TB Gold Plus among TB high-risk population in Bandung, Indonesia

**DOI:** 10.1128/jcm.01486-24

**Published:** 2025-03-07

**Authors:** Dewi Kartika Turbawaty, Syifa Nur Maulida, Darin Rizka Anadhea, Mulyanusa Amarullah Ritonga, Basti Andriyoko, Rudi Wisaksana, Agnes Rengga Indrati, Nanny Natalia Mulyani Soetedjo, Laniyati Hamijoyo, Raspati Cundarani Koesoemadinata, Bachti Alisjahbana, Ida Parwati

**Affiliations:** 1 Department of Clinical Pathology, Faculty of Medicine, Universitas Padjadjaran 106075 , Bandung, West Java, Indonesia; 2 Hasan Sadikin General Hospital, Bandung, West Java, Indonesia; 3 Research Center for Care and Control of Infectious Disease, Universitas Padjadjaran 61809 , Bandung, West Java, Indonesia; 4 Department of Obstetrics and Gynecology, Faculty of Medicine, Universitas Padjadjaran 106075 , Bandung, West Java, Indonesia; 5 Department of Internal Medicine, Faculty of Medicine, Universitas Padjadjaran 106075 , Bandung, West Java, Indonesia; University of Manitoba, Winnipeg, Manitoba, Canada

**Keywords:** TB infection, diagnostic performance, *Mycobacterium tuberculosis*, immunology

## Abstract

Tuberculosis (TB) remains a global public health problem. The determination of tuberculosis infection (TBI) using interferon-gamma release assay has now been used widely. We aim to evaluate the positivity rates of Standard E TB-Feron enzyme-linked immunosorbent assay (TB-Feron ELISA) and Standard F TB-Feron fluorescent immunoassay (TB-Feron FIA) and their agreement with QuantiFERON-TB Gold Plus (QFT-Plus) among TB high-risk populations in Bandung City, Indonesia. We conducted a cross-sectional study, including people with a high risk of acquiring TB. We screened subjects for TB symptoms and offered chest X-ray (CXR). Anyone with cough or CXR suggestive of TB was asked to give sputum samples for GeneXpert MTB/RIF Ultra test. The positivity rates and corresponding 95% confidence intervals (CI) were calculated among patients with bacteriologically confirmed TB and among patients with no evidence of TB, no history of TB, and no known contact with TB patient (low risk of TBI). The agreement with QFT-Plus was calculated using Cohen’s κ score. We enrolled 527 subjects, and the proportion of positive results among bacteriologically confirmed TB patients were 8 (53.3%; 95% CI 26.6–78.7), 9 (60.0%, 95% CI 32.3–83.7), and 10 (66.7%, 95% CI 38.4–88.8) by TB-Feron FIA, TB-Feron ELISA and QFT-Plus. The agreement between TB-Feron FIA and QFT-Plus among all subjects was similar to that of TB-Feron ELISA and QFT-Plus (84.1%, κ = 0.66, 95% CI 0.59–0.72). TB-Feron FIA and TB-Feron ELISA showed an acceptable clinical performance compared with QFT-Plus. These tests are useful alternatives for detecting TB infection.

**IMPORTANCE:**

This study evaluates the performance of two alternative interferon-gamma release assays, Standard E TB-Feron enzyme-linked immunosorbent assay and Standard F TB-Feron fluorescent immunoassay, for diagnosing tuberculosis infection (TBI) among high-risk populations in Bandung, Indonesia. Both assays demonstrated comparable clinical performance to the widely used QuantiFERON-TB Gold Plus (QFT-Plus). Given the global burden of tuberculosis, particularly in resource-limited settings, these findings suggest that the TB-Feron assays could serve as reliable alternatives to QFT-Plus for TBI detection. This research highlights the potential for these assays to improve tuberculosis diagnosis, offering a more accessible and efficient screening tool, especially for high-risk populations, and supporting broader tuberculosis surveillance.

## INTRODUCTION

Tuberculosis (TB) remains a global public health problem. The incidence, prevalence, and mortality rates of TB are gradually decreasing, but the absolute number of patients has been increasing annually (1, 2). Approximately 5 to 10% of people infected with *Mycobacterium tuberculosis* (Mtb) develop the disease during the first 2 to 5 years after infection (1). Progression to TB disease is postulated to be due to a series of pathogen factors and host mechanisms, including the innate immune response (3–5). Patients can develop a persistent immune response to Mtb antigens without clinical evidence of active disease. It is estimated that a quarter of the world’s population could be infected with Mtb (6).

Indonesia is a country with the second largest TB burden globally (prevalence estimate 647/100,000 population) (7). TB preventive therapy (TPT) is a strategy for controlling by providing medication to protect individuals with latent TB infection (TBI) or those at high risk of acquiring TB, as recommended by the World Health Organization (8). People with TBI are those with no evidence of active TB disease but tested positive for tuberculin skin test or interferon (IFN)-gamma release assay (IGRA) (9, 10). Treating high-risk individuals with TBI is an effective way to prevent TB. This involves administering a combination of medications and preventing the development of active TB (8).

The QuantiFERON-TB Gold Plus (QFT-Plus; Qiagen, Hilden, Germany) is one of the IGRAs known to be used. QFT-Plus employs TB-specific synthetic peptide antigens early secreted antigenic target 6 (ESAT-6) and culture filtrate protein 10 (CFP-10). Recently, the Standard E TB-Feron enzyme-linked immunosorbent assay (TB-Feron ELISA) (TB-Feron; SD Biosensor, Gyeonggi-do, Republic of Korea) was marked by European conformity and approved by the Ministry of Food and Drug Safety of the Republic of Korea for the detection of TBI. The TB antigen tube contains whole recombinant proteins of ESAT-6, CFP-10, and tuberculosis antigen (TB7.7). Using whole proteins instead of peptides increases the sensitivity of the assay because when the proteins are degraded into diverse small peptides, multiple epitopes may be present, thus stimulating T cells more effectively and potentially yielding higher IFN-γ values (11). Simplified versions of IGRAs are emerging, and one such test is the Standard F TB-Feron fluorescent immunoassay (TB-Feron FIA) (IFN-γ; SD Biosensor; Gyeonggi-do, Republic of Korea). These tests require less manual handling than ELISA-based IGRAs, and the results are available in 15 min–20 min once the 16 h–24 h incubation is completed. Additionally, TB-Feron FIA and TB-Feron ELISA require a lower volume of blood sample (3 mL) and allow a higher number of tests with 28 tests per 96-well plate compared to QFT-Plus which requires 4 mL blood sample with 22 tests per plate. These features enhance efficiency and reduce resource consumption in the testing process (12).

This study aims to evaluate the positivity rates of TB-Feron ELISA and TB-Feron FIA and their agreement with QFT-Plus.

## MATERIALS AND METHODS

### Study design and participants

A cross-sectional study was conducted in Bandung City, Indonesia, between June 2023 to February 2024. We consecutively enrolled people with a high risk of acquiring TB: healthcare workers (HCWs), pediatrics (aged ≥5 to ≤18 years old), people with a history of drug addiction (PHDAs), pregnant women, people living with HIV (PLWHs), people living with diabetes mellitus (PLWDs), and patients under immunosuppressive therapy. All subjects were included in this study based on previous studies showing increasing evidence of TBI and active TB among these groups (13).

The recruitment took place at the Research Center for Care and Control of Infectious Disease, Universitas Padjadjaran, Garuda Public Health Centre, Prison Class II Banceuy, and Dr. Hasan Sadikin General Hospital. Potential study subjects were interviewed to estimate their exposure status to TB and then classified into four risk categories: risk 1: never had any contact with TB patients; risk 2: had a history of contact with TB patients; risk 3: diagnosed with TB or under TB treatment; risk 4: had a history of TB treatment. Study subjects were then recruited consecutively and purposefully to reach a good proportion of patients in each category.

The IGRA tests were performed at the Biomedical Laboratory, Immunology Division, Faculty of Medicine, Universitas Padjadjaran. Samples were excluded if they were contaminated, hemolyzed, frozen, or did not follow the correct procedure during sampling.

### Study procedure

Data were collected using a case report form. All subjects were interviewed for the following information: age, gender, Bacillus Calmette-Guerin (BCG) vaccination status and scars, HIV diagnosis, history of TB, history of close contact with TB, history of TPT, and signs and symptoms of TB. Physical examination data were also collected on all subjects, and a chest X-ray (CXR) was offered, except to subjects who were recruited in the prison setting (PHDAs), because there was no X-ray machine in the prison clinic. Anyone with a productive cough or CXR suggestive of TB was asked to give two sputum samples for the GeneXpert MTB/RIF test. All subjects had blood drawn for the IGRA examination. Subjects who were diagnosed with TB during screening were re-categorized into the risk 3 group.

Based on all examination results and treatment decision, subjects were then classified into five categories: no TB, possible TB, clinically diagnosed TB, bacteriologically confirmed TB, and unclassified (Table 1).

**TABLE 1:** TB categorization^*a*^

TB category	Symptoms	Chest X-ray	Laboratory examinations	Treatment decision
No TB	Any symptoms or none	Normal or abnormal not related to TB	Negative results or not available	Not treated
Possible TB	Any symptoms or none	Abnormal suggestive of TB	Negative results or not available	Not treated
Clinically diagnosed TB	Any symptoms or none	Any results	Positive results of non-bacteriological examinations (urine LAM, FNAB) or not available	Treated
Bacteriologically confirmed TB	Any symptoms or none	Any results	Positive results of bacteriology examinations (GeneXpert, AFB)	Treated
Unclassified	Any symptoms or none	Refused/not available	Refused/not available	Not treated/not available

^*a*^ AFB, acid-fast bacilli; FNAB, fine needle aspiration biopsy; LAM, lipoarabinomannan; TB, tuberculosis.

### Test procedure

#### Standard F TB-Feron FIA

TB-Feron FIA uses specialized blood collection tubes, including a Nil tube, a TB antigen tube, and a mitogen tube. The Nil tube adjusts for the background IFN-γ level of the specimen. The TB antigen tube contains TB-specific recombinant protein antigens (ESAT-6, CFP-10, and TB7.7) to assess IFN-γ responses in T cells from individuals infected with *M. tuberculosis*, but generally not from uninfected or BCG-vaccinated people without disease or risk for latent TBI. The mitogen tube can be used as a positive control. This tube may also serve as a control for correct blood handling and incubation.

The test is performed in two stages. First, whole blood is collected into each of the blood collection tubes; after collection, the tubes were incubated at 37°C for 16 to 24 h, after which plasma is harvested and tested for the presence of IFN-γ produced in response to the recombinant protein antigens. IFN-γ quantification was processed with the Standard F2400 equipment (SD Biosensor, Gyeonggi-do, Republic of Korea), an automated FIA device. From sample loading into the cartridge to fluorescence intensity reading and IFN-γ quantification, followed by interpretation, the test is performed automatically within 15 min.

#### Standard E TB-Feron ELISA

TB-Feron ELISA uses specialized blood collection tubes, which are antigen-sensitized. Incubation of the blood occurs in the tubes for 16 to 24 h, after which plasma is harvested and tested for the presence of IFN-γ produced in response to the peptide antigens. The test is performed in two stages. First, whole blood is collected into each of the blood collection tubes, which include a Nil tube, a TB antigen tube, and a mitogen tube. The Nil tube adjusts for the background IFN-γ level of the sample. The TB antigen tube contains TB-specific recombinant protein antigens (ESAT-6, CFP-10, and TB7.7) to assess IFN-γ responses in T cells from individuals infected with Mtb, but generally not from uninfected or BCG-vaccinated people without disease or risk for TBI. The mitogen tube can be used with the test as a positive control. This tube may also serve as a control for correct blood handling and incubation. These three tubes should be incubated at 37°C as soon as possible and within 16 h of blood collection. Following the 16 to 24 h incubation period, the tubes are centrifuged, the plasma is collected, and the amount of IFN-γ (IU/mL) is measured by ELISA.

#### QuantiFERON-TB Gold Plus

QFT-Plus is an *in vitro* diagnostic test simulating ESAT-6 and CFP-10 proteins using a peptide cocktail to stimulate cells in heparinized whole blood. The QFT-Plus assay uses specialized blood collection tubes to collect whole blood. Incubation of the blood occurs in the tubes for 16 to 24 h, after which plasma is harvested and tested for the presence of IFN-γ produced in response to the peptide antigens. The QFT-Plus test is performed in two stages. First, whole blood is collected into each QFT-Plus Blood Collection Tube, including a Nil tube, a TB1 tube, a TB2 tube, and a mitogen tube. Alternatively, blood may be collected in a single generic tube containing lithium heparin as the anticoagulant and then transferred to QFT-Plus tubes. The mitogen tube is used with the QFT-Plus test as a positive control. This may be important where there is doubt about the individual’s immune status. The mitogen tube also serves as a control for correct blood handling and incubation.

The interpretations of Standard F TB-Feron FIA (14), Standard E TB-Feron ELISA (15), and QFT-Plus (16) were interpreted as positive when the IFN-γ value of the TB antigen tube minus that of the nil tube, referred to as the TB antigen-minus-Nil value, was ≥0.35 IU/mL and ≥25% of Nil value, with the Nil value ≤8.0 IU/mL. The results were negative when TB antigen-minus-Nil was <0.35 or TB antigen-minus-Nil was ≥0.35 but <25% of Nil value, with Nil value ≤8.0 IU/mL. The results were indeterminate when the Nil value was >8 IU/mL regardless of the TB antigen-minus-Nil value, or when TB antigen-minus-Nil was <0.35 and mitogen-minus-Nil was <0.5 with Nil value ≤8.0 IU/mL, or when TB antigen-minus-Nil was ≥0.35 IU/mL and <25% of Nil value, with Nil value ≤8.0 IU/mL (Table 2).

**TABLE 2:** Comparison of Standard F TB-Feron FIA, Standard E TB-Feron ELISA, and QuantiFERON-TB Gold Plus (14–16)^*a*^

	Standard F TB-Feron FIA	Standard E TB-Feron ELISA	QuantiFERON-TB Gold Plus
Number of tubes	Three: TB antigen tube, mitogen (positive control), Nil (negative control)	Three: TB antigen tube, mitogen (positive control), Nil (negative control)	Four: TB1, TB2, mitogen (positive control), Nil (negative control)
TB antigen tube contents	TB antigen tube: TB-specific recombinant protein antigens: ESAT-6, CFP-10, and TB7.7 Mitogen: non-TB-specific antigen Nil: empty tube	TB antige tube: TB-specific recombinant protein antigens: ESAT-6, CFP-10, and TB7.7 Mitogen: non-TB-specific antigen Nil: empty tube	TB1: peptides from ESAT-6 and CFP-10 designed to elicit cell-mediated immune responses from CD4+ T-helper lymphocytes TB2: set of peptides targeted to induce cell-mediated immune responses from CD8+ cytotoxic T lymphocytes Nil: empty tube
Allowable time from collection to incubation	16 h	16 h	16 h
Temperature requirements pre-incubation	15°C–25°C	15°C–25°C	Blood directly drawn into the QFT-Plus tubes: 22°C ± 5°C Blood collected into heparin tube: 17°C–25°C.
Interpretation parameters	Interpretation can be based on Nil, TB antigen, and mitogen quantitative results or only Nil and TB antigen quantitative results.	Interpretation can be based on Nil, TB antigen, and mitogen quantitative results or only Nil and TB antigen quantitative results.	Interpretation is based on the relative concentration of IFN-γ in the positive, negative, and TB antigen tubes.

^*a*^ CFP-10, culture filtrate protein 10; ESAT-6, early secreted antigenic target 6; FIA, fluorescent immunoassay; QFT-Plus, QuantiFERON-TB Gold Plus.

### Statistical analysis

Demographic and clinical characteristics were reported as mean and standard deviations (SD) or median and interquartile range (IQR) for numerical variables as appropriate according to the data distribution. Categorical variables were presented as frequencies and percentages.

The positivity rates were calculated among bacteriologically confirmed TB patients and among patients with no evidence of TB during examination, no history of TB, and no known contact with TB patients (low risk of TBI). The positivity rates were presented as percentages, and their 95% confidence intervals (CI) were also calculated using binomial exact test for proportions.

The agreement was comparing the qualitative results of TB-Feron ELISA and TB-Feron FIA to QFT-Plus. The concordance between TB-Feron FIA, TB-Feron ELISA, and QFT-Plus was measured using Cohen’s κ value: very low (≤0.2), low (0.2 to ≤0.4), medium (0.4 to ≤0.6), high (0.6 to ≤0.8), and very high (>0.8). Corresponding 95% CI for the κ values were calculated using an analytical method in the case of dichotomous variables (17). All statistical analyses were performed using Stata version 13 (StataCorp, College Station, TX, USA).

## RESULTS

### Subject characteristics

In this study, a total of 527 individuals were enrolled: 64 (12.1%) HCWs, 89 (16.9%) pregnant women, 101 (19.2%) pediatrics patients, 40 (7.6%) PHDAs, 100 (18.9%) PLWHs, 101 (19.2%) PLWDs, and 32 (6.1%) immunocompromised patients, of whom 30 (93.7%) had systemic lupus erythematosus and two (6.3%) had rheumatoid arthritis. The median age of all subjects was 32 years old (IQR 23–44), and 235 (44.6%) were males. There were 134 (25.4%) people with a history of TB and 166 (31.5%) with a history of contact with TB patients. As many as 368 (69.8%) had BCG scar, and only 64 (12.1%) had TPT, mostly PLWHs. Almost one-third (164, 31.1%) of subjects were smokers, and 119 (22.6%) were coughing during baseline examination.

Excluding PHDAs and participants who refused CXR, there were 89 (16.9%) patients with suggestive active TB in their CXR reading. As many as 14 (2.7%) had a positive GeneXpert MTB/Rif result. Among PLWHs, five (5.0%) had positive urine lipoarabinomannan (LAM), one patient had positive acid-fast bacilli (AFB), and one patient had positive fine needle aspiration biopsy (FNAB). Of all subjects, 23 (4.4%) were diagnosed with TB clinically, of whom 9 (9.0%) were PLWHs, 7 (6.9%) were pediatrics, 2 (2.0%) were PLWDs, 2 (6.3%) were immunocompromised patients, 1 (2.5%) was a PHDA, 1 (1.5%) was an HCW, and 1 (1.1) was a pregnant woman. Of all subjects, 251 (47.6%) were categorized into risk 1, 128 (24.3%) were in risk 2, 44 (8.4%) were in risk 3, and 104 (19.7%) were in risk 4. According to TB category, 312 (59.2%) had no TB, 80 (15.2%) were categorized as possible TB, 29 (5.5%) were clinically diagnosed TB, 15 (2.8%) were bacteriologically confirmed TB, and 91 (17.3%) were unclassified (Table 3).

**TABLE 3:** Characteristics of study subjects^*a*^

Characteristics	Total (*n* = 527)	HCWs (*n* = 64)	Pregnant women (*n* = 89)	Pediatrics patients (*n* = 101)	PHDAs (*n* = 40)	PLWHs (*n* = 100)	PLWDs (*n* = 101)	Immunocompromised (*n* = 32)
Male	235 (44.6)	14 (21.9)	0 (0.0)	57 (56.4)	40 (100)	77 (77.0)	46 (45.5)	1 (3.1)
Median age (IQR)	32 (23–44)	30 (26–37)	25 (18–34)	11 (8–14)	32 (28–40)	38.5 (31-45)	57 (51–65)	32.5 (28-42)
History of TB	134 (25.4)	10 (15.6)	18 (17.8)	11 (12.3)	2 (5.0)	58 (58.0)	24 (23.8)	11 (34.4)
History of TB contact	166 (31.5)	26 (40.6)	25 (31.3)	46 (45.5)	3 (7.5)	19 (19.0)	39 (38.6)	8 (25.0)
BCG scar	368 (69.8)	57 (89.1)	70 (78.7)	92 (91.1)	37 (80.0)	62 (62.0)	84 (83.2)	23 (71.9)
History of TPT	64 (12.1)	6 (9.4)	7 (7.9)	7 (17.8)	2 (5.0)	30 (30.0)	0 (0.0)	12 (37.5)
HIV status								
Negative	183 (34.7)	40 (62.5)	83 (93.3)	13 (12.8)	32 (80.0)	0 (0.0)	10 (9.9)	9 (28.1)
Positive	100 (19.0)	0 (0.0)	0 (0.0)	0 (0.0)	0 (0.0)	100 (100.0)	0 (0.0)	0 (0.0)
Unknown	237 (45.0)	24 (37.5)	6 (6.7)	88 (87.2)	8 (20.0)	0 (0.0)	91 (90.1)	23 (71.9)
Smoking	164 (31.1)	2 (3.1)	63 (70.8)	5 (4.9)	37 (92.5)	34 (34.0)	19 (18.8)	4 (12.5)
Coughing	119 (22.6)	4 (6.3)	41 (46.1)	26 (25.7)	5 (12.5)	17 (17.0)	20 (19.8)	6 (18.8)
BMI* (kg/m^2^)								
Underweight	79 (15.0)	6 (9.4)	17 (19.1)	19 (18.8)	3 (5.0)	29 (29.0)	3 (3.0)	2 (6.3)
Normal	239 (45.4)	13 (20.3)	40 (44.9)	72 (71.3)	22 (55.0)	46 (46.0)	32 (31.7)	14 (43.8)
Overweight	87 (16.5)	19 (29.7)	11 (12.4)	8 (7.9)	6 (15.0)	13 (13.0)	24 (23.8)	6 (18.8)
Obese	122 (23.1)	26 (40.6)	21 (23.6)	2 (2.0)	9 (22.5)	12 (12.0)	42 (41.6)	10 (31.3)
Chest X-ray								
Normal	230 (43.6)	54 (84.4)	20 (22.5)	43 (42.5)	0 (0.0)	67 (67.0)	28 (27.7)	18 (56.3)
Suggestive of active TB	89 (16.9)	4 (6.3)	16 (18.0)	40 (39.6)	0 (0.0)	8 (8.0)	20 (19.8)	1 (3.1)
Suggestive of old TB	10 (1.9)	0 (0.0)	1 (1.1)	1 (0.9)	0 (0.0)	3 (3.0)	4 (4.0)	1 (3.1)
Refused	63 (12.0)	0 (0.0)	45 (50.6)	12 (11.8)	0 (0.0)	1 (1.0)	5 (5.0)	0 (0.0)
Abnormal non-TB related**	95 (18.0)	6 (9.4)	7 (7.9)	5 (4.9)	0 (0.0)	21 (21.0)	44 (43.5)	12 (37.5)
GeneXpert MTB/Rif positive	14 (2.7)	3 (4.6)	0 (0.0)	0 (0.0)	1 (2.5)	6 (6.0)	3 (3.0)	1 (3.1)
Urine LAM positive	5 (0.9)	0 (0.0)	0 (0.0)	0 (0.0)	0 (0.0)	5 (5.0)	0 (0.0)	0 (0.0)
AFB positive	1 (0.2)	0 (0.0)	0 (0.0)	0 (0.0)	0 (0.0)	1 (1.0)	0 (0.0)	0 (0.0)
FNAB positive	1 (0.2)	0 (0.0)	0 (0.0)	0 (0.0)	0 (0.0)	1 (1.0)	0 (0.0)	0 (0.0)
Clinical TB	23 (4.4)	1 (1.5)	1 (1.1)	7 (6.9)	1 (2.5)	9 (9.0)	2 (2.0)	2 (6.3)
Risk category								
Risk 1 never had TB contact	251 (47.6)	30 (46.9)	48 (53.9)	44 (43.6)	34 (85.0)	34 (34.0)	45 (44.6)	16 (50.0)
Risk 2 had history of TB contact	128 (24.3)	20 (31.2)	29 (32.6)	32 (31.7)	2 (5.0)	8 (8.0)	32 (31.7)	5 (15.6)
Risk 3 under TB treatment	44 (8.4)	4 (6.2)	1 (1.1)	7 (6.9)	2 (5.0)	22 (22.0)	5 (5.0)	3 (9.4)
Risk 4 had history of past TB	104 (19.7)	10 (15.6)	11 (12.4)	18 (17.8)	2 (5.0)	36 (36.0)	19 (18.8)	8 (25.0)

^*a*^ All data were presented as numbers and percentages unless stated otherwise. *BMI for adults was classified as underweight (<18.5), normal (18.5–22.9), overweight (23.0–24.9), and obese (≥25.0), BMI for pediatrics was classified by age; BMI for children aged 5–18 years was classified as underweight (−3 SD to <−2 SD), normal (–2 SD to +1 SD), overweight (+1 SD to +2 SD), and obese (>+2 SD). **Abnormal non-TB related was defined when chest X-ray results showed cardiomegaly, pleural effusion, atherosclerosis aorta, or other abnormalities that do not relate to TB. GeneXpert was performed if the patient had symptoms of cough and/or had chest X-ray results suggestive of active/old TB. Abbreviations: AFB, acid-fast bacilli; BCG, Bacillus Calmette-Guerin; BMI, body mass index; FNAB, fine needle aspiration biopsy; HCWs, healthcare workers; HIV, human immunodeficiency virus; IQR, interquartile range; PHDAs, people with history of drug addiction; PLWHs, people living with HIV; PLWDs, people living with diabetes mellitus; TB, tuberculosis; TPT, TB preventive therapy.

### TB-Feron FIA, TB-Feron ELISA, and QFT-Plus results in all subjects

Table 4 shows the result of each IGRA assay using TB-Feron FIA, TB-Feron ELISA, and QFT-Plus. Among all subjects, 39.3% had a positive result by TB-Feron FIA, 41.2% were positive by TB-Feron ELISA, and 31.1% were positive by QFT-Plus.

**TABLE 4:** Results of TB-Feron FIA, TB-Feron ELISA, and QFT-Plus^*a*^

IGRA results	Total (*n* = 527)	HCWs (*n* = 64)	Pregnant women (*n* = 89)	Pediatrics (*n* = 101)	PHDAs (*n* = 40)	PLWHs (*n* = 100)	PLWDs (*n* = 101)	Immunocompromised (*n* = 32)
TB-Feron FIA, n (%)								
Positive	207 (39.3)	30 (46.9)	29 (32.6)	27 (26.7)	27 (67.5)	37 (37.0)	48 (47.5)	9 (28.1)
Negative	309 (58.6)	34 (53.1)	58 (65.2)	72 (71.3)	13 (32.5)	59 (59.0)	50 (49.5)	23 (71.9)
Indeterminate	11 (2.1)	0 (0.0)	2 (2.2)	2 (2.0)	0 (0.0)	4 (4.0)	3 (3.0)	0 (0.0)
TB-Feron ELISA, *n* (%)								
Positive	217 (41.2)	23 (35.9)	35 (39.3)	30 (29.7)	25 (62.5)	41 (41.0)	54 (53.5)	9 (28.1)
Negative	307 (58.3)	41 (64.1)	54 (60.7)	71 (70.3)	15 (37.5)	57 (57.0)	46 (45.5)	23 (71.9)
Indeterminate	3 (0.5)	0 (0.0)	0 (0.0)	0 (0.0)	0 (0.0)	2 (2.0)	1 (1.0)	0 (0.0)
QFT-Plus, *n* (%)								
Positive	164 (31.1)	18 (28.1)	26 (29.2)	22 (21.8)	22 (55.0)	34 (34.0)	37 (36.6)	5 (15.6)
Negative	361 (68.5)	46 (71.9)	62 (69.7)	79 (78.2)	18 (45.0)	66 (66.0)	63 (62.4)	27 (84.4)
Indeterminate	2 (0.4)	0 (0.0)	1 (1.1)	0 (0.0)	0 (0.0)	0 (0.0)	1 (1.0)	0 (0.0)

^*a*^ ELISA, enzyme-linked immunosorbent assay; FIA, fluorescent immunoassay; HCWs, healthcare workers; PHDAs, people with history of drug addiction; PLWDs, people living with diabetes mellitus; PLWHs, people living with human immunodeficiency virus.

Among HCWs, the positivity rate on TB-Feron FIA, TB-Feron ELISA, and QFT-Plus were 46.9%, 35.9%, and 28.1%, respectively. Among pediatrics, the proportion of positive results was 26.7%, 29.7%, and 21.8% by TB-Feron FIA, TB-Feron ELISA, and QFT-Plus, respectively. The positivity rate among pregnant women was TB-Feron FIA (32.6%), TB-Feron ELISA (39.3%), and QFT-Plus (29.2%). IGRA positives among PHDAs were 67.5%, 62.5%, and 55.0% from TB-Feron FIA, TB-Feron ELISA, and QFT-Plus, respectively.

Among PLWHs, 37.0% had positive results by TB-Feron FIA, 41.0% were positive using TB-Feron ELISA, and only 34.0% were positive by QFT-Plus. Among PLWDs, about half were IGRA positive by TB-Feron FIA (47.5%) and TB-Feron ELISA (53.5%), but only 36.6% were IGRA positive when using QFT-Plus. The positivity rates among people with immunosuppressive conditions were 28.1%, 28.1%, and 15.6%, respectively, using TB-Feron FIA, TB-Feron ELISA, and QFT-Plus.

The highest proportions of indeterminate result were found when using TB-Feron FIA 11 (2.1%), followed by TB-Feron ELISA 3 (0.5%) and QFT-Plus 2 (0.4%). The proportion of indeterminate results were highest in the PLWHs (4.0%), followed by the PLWD group (3.0%), pregnant women (2.2%), and pediatrics (2.2%) when using TB-Feron FIA. The proportion of indeterminate results was lower among PLWHs (2.0%) and PLWDs (1.0%) when using TB-Feron ELISA. Only two patients from PLWDs and a pregnant woman had an indeterminate result when using QFT-Plus.

### Positivity rates of TB-Feron FIA, TB-Feron ELISA, and QFT-Plus among bacteriologically confirmed TB patients and among subjects with low risk of TBI

There were 15 bacteriologically confirmed patients (Table 5). PLWHs had the highest number of patients (*n* = 7), followed by PLWDs (*n* = 3), HCWs (*n* = 3), immunocompromised patients (*n* = 1), and PHDAs (*n* = 1). Of 15 patients, positive results were 8 (53.3%, 95% CI 26.6–78.7), 9 (60.0%, 95% CI 32.3–83.7), and 10 (66.7%, 95% CI 38.4–88.8) by TB-Feron FIA, TB-Feron ELISA, and QFT-Plus, respectively.

**TABLE 5:** Characteristics of bacteriologically confirmed TB patients and their IGRA results^*a*^

No.	Patient group	Age	Gender	BMI (kg/m^2^)	Chest X-ray	GeneXpert date	GeneXpert result	Sampling date	TB-Feron FIA result	TB-Feron ELISA result	QFT-Plus result
1	HCWs	44	Female	16.4	Suggestive of active TB	14 Jul 2022^*a*^	Mtb detected, Rif resistant	9 Jan 2024	Negative	Negative	Negative
2	HCWs	37	Female	21.6	Abnormal-not TB	24 May 2023	Mtb detected, Rif susceptible	23 Nov 2023	Positive	Negative	Positive
3	HCWs	55	Female	21.1	Suggestive of active TB	10 Aug 2023	Mtb detected, Rif susceptible	7 Nov 2023	Positive	Positive	Positive
4	PHDAs	33	Male	16.3	Refused	15 Feb 2024	Mtb detected, Rif susceptible	19 Feb 2024	Negative	Negative	Negative
5	PLWHs	33	Male	18.7	Abnormal-not TB	30 May 2023	Mtb detected, Rif susceptible	31 Aug 2023	Negative	Positive	Positive
6	PLWHs	34	Male	22.8	Suggestive of active TB	15 Jun 2023	Mtb detected, Rif susceptible	6 Sep 2023	Negative	Positive	Positive
7	PLWHs	19	Female	16.9	Suggestive of active TB	19 Jun 2023	Mtb detected, Rif susceptible	12 Sep 2023	Positive	Positive	Negative
8	PLWHs	31	Male	18.7	Abnormal-not TB	27 May 2023 (AFB date)	GeneXpert not done, but AFB positive 1	31 Aug 2023	Positive	Positive	Positive
9	PLWHs	46	Male	21.7	Normal	19 Jun 2023	Mtb detected, Rif susceptible	14 Sep 2023	Positive	Positive	Positive
10	PLWHs	27	Male	19.6	Suggestive of old TB	4 Apr 2023	Mtb detected, Rif susceptible	12 Sep 2023	Negative	Negative	Negative
11	PLWHs	24	Male	20.2	Suggestive of active TB	29 Jun 2023	Mtb detected, Rif susceptible	7 Sep 2023	Indeterminate	Positive	Negative
12	PLWDs	51	Female	28.1	Refused	17 Sep 2023	Mtb detected, Rif susceptible	6 Nov 2023	Negative	Negative	Positive
13	PLWDs	73	Male	18.9	Suggestive of active TB	13 Sep 2023	Mtb detected, Rif susceptible	14 Nov 2023	Positive	Negative	Positive
14	PLWDs	66	Female	18.7	Suggestive of active TB	18 Sep 2023	Mtb detected, Rif susceptible	14 Sep 2023	Positive	Positive	Positive
15	Immunocompromised	32	Female	18.5	Suggestive of active TB	31 Jul 2023	Mtb detected, Rif susceptible	11 Sep 2023	Positive	Positive	Positive

^*a*^ This patient had been under TB treatment for multidrug-resistant TB for 19 months. Abbreviations: AFB, acid-fast bacilli; BMI, body mass index; ELISA, enzyme-linked immunosorbent assay; FIA, fluorescent immunoassay; HCWs, healthcare workers; Mtb, *Mycobacterium tuberculosis;* PHDAs, people with history of drug addiction; PLWDs, people living with diabetes mellitus; PLWHs, people living with human immunodeficiency virus; QFT, QuantiFERON; Rif, rifampicin; TB, tuberculosis.

A patient who tested negative by TB-Feron FIA, TB-Feron ELISA, and QFT-Plus was an HCW diagnosed with multidrug-resistant TB and had been under treatment for 19 months (patient no. 1). Another patient tested negative by all IGRA assays was a PLWH who nearly finished his TB treatment (patient no. 10). Another patient tested negative by all IGRA assays was a PHDA, with 4 days gap between the Xpert date and blood sampling date (patient no. 4). This patient had a body mass index (BMI) of 16.3 kg/m^2^ (underweight) which may explain the lack of interferon-gamma production.

Four patients with negative TB-Feron FIA (patient nos. 5, 6, and 12) and indeterminate result (patient no.11) but positive on TB-Feron ELISA or QFT-Plus had 3 to 4 months gap between the GeneXpert date and blood sampling date. Two patients who tested negative on TB-Feron ELISA but positive on TB-Feron FIA or QFT-Plus had 6 and 2 months gap between GeneXpert date and blood sampling date (patient nos. 2 and 13). While two other patients who tested negative on QFT-Plus but positive or indeterminate by TB-Feron FIA and TB-Feron ELISA had 3 months gap between GeneXpert date and blood sampling date (patient nos. 7 and 11) (Table 5).

The number of subjects with no TB during examination, no history of TB, and no known contact with TB patient were 216, of whom 36 (16.7%) were HCWs, 17 (7.9%) were pregnant women, 25 (11.6%) were pediatric patients, 4 (1.8%) were PHDAs, 63 (29.2%) were PLWHs, 49 (22.7%) were PLWDs, and 22 (10.2%) were immunocompromised patients. The results of TB-Feron FIA, TB-Feron ELISA, and QFT-Plus among these subjects are described in Table 6.

**TABLE 6:** Results of TB-Feron FIA, TB-Feron ELISA, and QFT-Plus among subjects with low risk of TBI^*a*^

IGRA assay	IGRA result	Frequency, *n* = 216 (*n*)	Percentage (%)	95% CI (%)
TB-Feron FIA	Positive	71	32.7	26.6–39.6
	Negative	139	64.4	57.6–70.7
	Indeterminate	6	2.8	1.0–5.9
TB-Feron ELISA	Positive	73	33.8	27.5–40.5
	Negative	140	64.8	58.0–71.2
	Indeterminate	3	1.4	0.3–4.0
QFT-Plus	Positive	45	20.8	15.6–26.9
	Negative	170	78.7	72.6–84.0
	Indeterminate	1	0.5	0.01–2.5

^*a*^ Low risk of TBI: patients with no evidence of TB during examinations, no history of TB, and no known contact with TB patients. Abbreviations: CI, confidence interval; ELISA, enzyme-linked immunosorbent assay; FIA, fluorescent immunoassay; IGRA, interferon-gamma release assay; QFT, QuantiFERON.

The positivity rates among study participants in the low-risk TBI group were 32.7% (95% CI 26.6–39.6), 33.8% (95% CI 27.5–40.5), and 20.8% (95% CI 15.6–26.9) by TB-Feron FIA, TB-Feron ELISA, and QFT-Plus, respectively.

Table 7 shows the agreement rates and κ values of TB-Feron FIA and TB-Feron ELISA compared with QFT-Plus. We found high agreement in all groups for both TB-Feron FIA and TB-Feron ELISA among all subjects (84.1%, κ = 0.66, 95% CI 0.60–0.73). The agreement among HCWs between TB-Feron FIA and QFT-Plus was 81.2%, κ = 0.61, 95% CI 0.43–0.80, while agreement between TB-Feron ELISA and QFT-Plus was 89.1%, κ = 0.75, 95% CI 0.58–0.92. Among pregnant women, we found a high level of agreement between TB-Feron FIA and QFT-Plus (87.2%, κ = 0.71, 95% CI 0.55–0.87) and a slightly higher agreement between TB-Feron ELISA and QFT-Plus (87.5%, κ = 0.73, 95% CI 0.58–0.87). Among the pediatric group, we found a high agreement between TB-Feron FIA and QFT-Plus (87.9%, κ = 0.67, 95% CI 0.50–0.84) and a medium agreement between TB-Feron ELISA and QFT-Plus (84.2%, κ = 0.59, 95% CI 0.41–0.77). Among the PHDAs, we found a medium agreement between TB-Feron FIA and QFT-Plus (77.5%, κ = 0.53, 95% CI 0.28–0.79) and a high agreement between TB-Feron ELISA and QFT-Plus (82.5%, κ = 0.64, 95% CI 0.40–0.88).

**TABLE 7:** The agreement of TB-Feron FIA and TB-Feron ELISA with QFT-Plus stratified by the subject’s group, excluding indeterminate results^*a*^

IGRA result	QFT-Plus		Agreement (%)	κ Value (95% CI)
	Positive, *n* (%)	Negative, *n* (%)		
All subjects				
TB-Feron FIA (*n* = 515)				
Positive	144 (88.3)	63 (17.9)	84.1	0.66 (0.60–0.73)
Negative	19 (11.7)	289 (82.1)		
TB-Feron ELISA (*n* = 523)				
Positive	149 (90.8)	68 (18.9)	84.1	0.66 (0.60–0.73)
Negative	15 (9.1)	291 (81.1)		
HCWs				
TB-Feron FIA (*n* = 64)				
Positive	18 (100)	12 (26.1)	81.2	0.61 (0.43–0.80)
Negative	0 (0.0)	34 (73.9)		
TB-Feron ELISA (*n* = 64)				
Positive	17 (94.4)	6 (13.0)	89.1	0.75 (0.58–0.92)
Negative	1 (5.6)	40 (87.0)		
Pregnant women				
TB-Feron FIA (*n* = 86)				
Positive	22 (84.6)	7 (11.7)	87.2	0.71 (0.55–0.87)
Negative	4 (15.4)	53 (88.3)		
TB-Feron ELISA (*n* = 88)				
Positive	25 (96.2)	10 (16.1)	87.5	0.73 (0.58–0.87)
Negative	1 (3.8)	52 (83.9)		
Pediatrics				
TB-Feron FIA (*n* = 99)				
Positive	18 (85.7)	9 (11.5)	87.9	0.67 (0.50–0.84)
Negative	3 (14.3)	69 (88.5)		
TB-Feron ELISA (*n* = 101)				
Positive	18 (81.8)	12 (15.2)	84.2	0.59 (0.41–0.77)
Negative	4 (18.2)	67 (84.8)		
PHDAs				
TB-Feron FIA (*n* = 40)				
Positive	20 (90.9)	7 (38.9)	77.5	0.53 (0.28–0.79)
Negative	2 (9.1)	11 (61.1)		
TB-Feron ELISA (*n* = 40)				
Positive	20 (90.9)	5 (27.8)	82.5	0.64 (0.40–0.88)
Negative	2 (9.1)	13 (72.2)		
PLWHs				
TB-Feron FIA (*n* = 96)	27 (79.4)	10 (16.1)	82.3	0.62 (0.40–0.78)
Positive	7 (20.6)	52 (83.9)		
Negative				
TB-Feron ELISA (*n* = 98)				
Positive	30 (88.2)	11 (17.2)	84.7	0.68 (0.53–0.82)
Negative	4 (11.8)	53 (82.8)		
PLWDs				
TB-Feron FIA (*n* = 98)				
Positive	34 (91.9)	14 (23.0)	82.6	0.65 (0.51–0.80)
Negative	3 (8.1)	47 (77.0)		
TB-Feron ELISA (*n* = 100)				
Positive	34 (91.9)	20 (31.8)	77.0	0.55 (0.40–0.70)
Negative	3 (8.1)	43 (68.2)		
Immunocompromised				
TB-Feron FIA (*n* = 32)				
Positive	5 (100)	4 (14.8)	87.5	0.64 (0.34–0.95)
Negative	0 (0.0)	23 (85.2)		
TB-Feron ELISA (*n* = 32)				
Positive	5 (100)	4 (14.8)	87.5	0.64 (0.34–0.95)
Negative	0 (0.0)	23 (85.2)		

^*a*^ ELISA, enzyme-linked immunosorbent assay; FIA, fluorescent immunoassay; HCWs, healthcare workers; IGRA, interferon-gamma release assay; PHDAs, people with history of drug abuse; PLWDs, people living with diabetes mellitus; PLWHs, people living with HIV; TB, tuberculosis. Indeterminate results were excluded from the analysis.

A high agreement between TB-Feron FIA and QFT-Plus (82.3%, κ = 0.62, 95% CI 0.46–0.78) and between TB-Feron ELISA and QFT-Plus (84.7%, κ = 0.68, 95% CI 0.53–0.82) was also found among PLWHs. Among PLWDs, we found a high agreement of TB-Feron FIA and QFT-Plus (82.6%, κ = 0.65, 95% CI 0.51–0.80) and a medium agreement of TB-Feron ELISA and QFT-Plus (77.0%, κ = 0.55, 95% CI 0.40–0.70). Among immunocompromised patients, a high agreement of TB-Feron FIA and TB-Feron ELISA with QFT-Plus with the same percentage of agreement and κ value (87.5%, κ = 0.64, 95% CI 0.34–0.95) was determined.

## DISCUSSION

This study aims to determine the positivity rates of TB-Feron FIA and ELISA and the agreement of each IGRA compared to the QFT-Plus. Among 527 subjects, TB-Feron FIA and TB-Feron ELISA exhibit a higher positivity rate than QFT-Plus. This result was similar to Jung et al.’s findings, which comprised TB-Feron ELISA and QFT-Plus individuals with active TB infection, immunocompromised patients, and healthy controls with TBI (18). This may be because the TB-Feron FIA and TB-Feron ELISA feature more TB-specific recombinant whole protein antigens, specifically ESAT-6, CFP-10, and TB7.7, in contrast to the QFT-Plus, which only encompasses two protein peptide forms (ESAT-6 and CFP-10) with specifically stimulated CD4+ and CD8+ T cells. The use of whole recombinant proteins in TB-Feron assays may facilitate the degradation into a variety of smaller peptides, which can then present multiple epitopes on the surface of antigen-presenting cells. This multiepitope stimulation of T cells may enhance the overall sensitivity of the IGRA, potentially leading to higher IFN-γ levels and showing higher sensitivity (19, 20).

A low reaction to mitogen (<0.5 IU/mL) indicates an indeterminate result when a blood sample has a negative response to the TB antigens. The highest frequency of indeterminate results was found in TB-Feron FIA, followed by TB-Feron ELISA and QFT-Plus. The presence of indeterminate results in IGRA introduces a noteworthy limitation, which influences the concordance observed in this study. Indeterminate results signify that the test cannot definitively ascertain positivity or negativity. The findings may be attributed to a combination of biological and technical factors. Biological factors leading to indeterminate results may include inadequate mitogen response (positive control) due to immunodeficiency states or high Nil response (negative control) caused by elevated IFN-γ levels unrelated to TB antigen stimulation (21). Cellular immune dysfunction, which is caused by diminished T lymphocyte counts, may result in inadequate mitogen response. This is observed in conditions like HIV infection and cancer (21–24). Indeterminate results can occur in 2% of the healthy population and up to 12% of immunocompromised or immunosuppressed patients (25, 26). Indeterminate results can also be influenced by various other factors, including age, medication, and concurrent medical conditions (21). In immunocompromised populations, most indeterminate results are associated with underlying immunodeficiency (27).

The indeterminate results on TB-Feron FIA predominantly fell in a low mitogen response rather than a high Nil. One indeterminate result was observed in a pediatric subject who was underweight and presented with leukocytosis. The IFN-γ result observed in this patient may not be associated with a low BMI, but could potentially be linked to lymphocytopenia and the concomitant presence of bacterial or fungal infections, which could interfere with the immune response and contribute to the observed immune dysregulation (28, 29).

Three of the indeterminate results in PLWHs were attributed to low mitogen responses and one PLWH resulted in high Nil. One PLWH individual with low response of mitogen had CD4 counts <200 cells/µL, while the remaining three had CD4 counts ≥200 cells/µL (Table S1). Notably, all individuals with indeterminate results fell within the normal and obese BMI categories, suggesting that inadequate mitogen response in those with CD4 counts ≥200 cells/µL may be attributed to immunosenescence. HIV infection can induce alterations in adaptive immune mechanisms and immunosenescence due to sustained and chronic inflammation. HIV infection disrupts thymic architecture by impairing stromal organization, consequently interfering with thymopoiesis and prompting CD4+ T cell apoptosis (30, 31).

In immunocompromised populations such as PLWDs and pregnant women, most indeterminate results showed a low response of mitogen (Table S1) that might be associated with underlying immunodeficiency (27). In pregnant women, it is known that during pregnancy, the cytokines generated at the feto-maternal interface play a critical role in modulating immune responses, particularly by downregulating the production of proinflammatory cytokines, including IFN-γ (32). Furthermore, diabetes mellitus is known to be associated with lower levels of mycobacterial antigen-specific IFN-γ release (33). Unlike normal TB patients, studies have shown that tuberculosis-diabetes mellitus (TB-DM) patients exhibit a hyperinflammatory response to Mtb antigens, particularly in peripheral blood (34). This heightened response may be due to several factors. First, the increased Th1 and Th17 responses are mainly present in the peripheral blood of TB-DM patients, while anti-inflammatory responses that promote Mtb growth occur only in the lungs. Second, while proinflammatory cytokines like IFN-γ are produced at higher levels in the lungs of TB-DM patients (as seen in mice), they may not effectively activate macrophages or cytotoxic T cells to kill *M. tuberculosis* (35, 36). Lastly, this overactive immune response to mycobacterial antigens in TB-DM patients may result in an ineffective immune system response against *M. tuberculosis* and lead to indeterminate results in IGRA assays (Table S1) (34).

In contrast, a high level of Nil and mitogen was observed in one pediatric subject (Table S1). The high Nil level suggests a non-specific response to another heterophilic antigen unrelated to tuberculosis, while the elevated mitogen level indicates a TBI (21, 37, 38). This finding may be linked to a co-infection present at the time of recruitment, as the patient exhibited symptoms such as cough and fever, along with a chest X-ray that was indicative of TB. Furthermore, we still cannot exclude the preanalytical issues while the subjects did not show leukocytosis from the hematologic routine test. These indeterminate samples also were reanalyzed using the same sample and showed the same result. This finding suggests that preanalytical factors cannot be ruled out as a potential factor on the outcome of the patient samples, which could affect the reliability of the results.

Various technical factors also influenced the indeterminate results. Preanalytical issues such as delayed sample incubation or processing, inadequate tube homogenization, and improper blood volume are among the factors (24). This study involved qualified personnel who conducted examinations and ensured consistent handling of all samples throughout the preanalytical phase. This approach effectively minimized the potential preanalytical errors, except those inherent to the samples. Analytical factors contributing to indeterminate results in this study include variations in IFN-γ quantification methods between the two methodologies. QFT-Plus employs an ELISA technique with enzyme and substrate reactions, where examination outcomes are expressed as optical density and subsequently converted to international units (IU/mL) through standard curve calibration. In contrast, the TB-Feron FIA uses a fluorescent immunoassay method, where the sample reacts with reagents on the conjugate pad and then migrates along the cartridge membrane. Fluorescence intensity on the membrane is then automatically quantified by the Standard F analyzer.

Among people who are at a high risk of acquiring TB, we found a high agreement between TB-Feron FIA and QFT-Plus, as well as a high agreement between TB-Feron ELISA and QFT-Plus. Although the agreement between TB-Feron FIA and TB-Feron ELISA with QFT-Plus across all groups was high, the highest level of agreement was found in HCWs between TB-Feron ELISA and QFT-Plus. Studies involving HCWs and TB patients have reported a concordance rate of 92.0% and an overall agreement of 85.9% between the TB-Feron ELISA and the QFT-Plus assays (39, 40). Additionally, Lee et al. found the TB-Feron enzyme-linked immunosorbent assay and QFT-Plus demonstrated a concordance rate of 91%, which is slightly higher than the concordance observed in our study (41).

There is no gold standard for TBI, and the nature of IGRA as an immunological biomarker instead of a microbiological test makes it difficult to compare. However, we found relatively high proportions of positive results among bacteriologically confirmed TB patients by these tests. However, these patients may have already converted to IGRA negative during TB treatment (42, 43).

The proportions of positive results among participants with no TB, no history of TB, and no known contact with TB patients reflect the prevalence of TBI among these TB high-risk populations in TB endemic country. People who are at low risk of TBI group may have been in contact with TB patients without their knowledge (32, 33).

This is the first study that compares TB-Feron ELISA and TB-Feron FIA with QFT-Plus, with a relatively large sample size in TB endemic settings. This is also the first study that includes pregnant women, pediatrics patients, PHDAs, immunocompromised patients, PLWDs, and PLWHs.

The study encountered limitations when evaluating the performance of different IGRA assays. There are gaps between the date of examination for TB diagnosis (GeneXpert) and blood withdrawals for the IGRA assays. Naturally, TB infection is not easy to diagnose because the initial test relies on the subject’s immune response and must be followed by a CXR to rule out active pulmonary TB. People with active TB disease can also result in negative for IGRA if the immune system is disturbed. The potential misclassification of PHDA cases that should be classified as TB is a concern, particularly due to the absence of CXR. Also, the analytical issues associated with the TB-Feron FIA may have led to inconsistencies in the fluorescence signal, which resulted in indeterminate outcomes. To improve the reliability and accuracy of the assay, it is important to address these variables in future studies. Moreover, the study was conducted in a country with suboptimal availability of individual health data, which makes it challenging to obtain accurate information regarding patient contact with TB cases.

### Conclusion

The high agreement of the TB-Feron FIA and TB-Feron ELISA showed a comparable and acceptable clinical performance for detecting TBI to that of QFT-Plus in different groups. Therefore, TB-Feron FIA and TB-Feron ELISA would be useful alternatives to current IGRAs, such as the QFT-Plus, for detecting TBI.

## SUPPLEMENTAL MATERIAL

Table S1 jcm.01486-24-s0001.docx Quantitative values of indeterminate results of TB-Feron FIA, TB-Feron Elisa, and QFT-Plus.

ASM does not own the copyrights to Supplemental Material that may be linked to, or accessed through, an article. The authors have granted ASM a non-exclusive, world-wide license to publish the Supplemental Material files. Please contact the corresponding author directly for reuse.
