# Supplementary material for: Standard E TB-Feron ELISA and Standard F TB-Feron FIA positivity rates and agreement with QuantiFERON-TB Gold Plus among TB high-risk population in Bandung, Indonesia
Source: J Clin Microbiol. 2025 Mar 7;63(4):e01486-24. doi: 10.1128/jcm.01486-24 (PMC11980364; doi:10.1128/jcm.01486-24)
Supplement: Table S1 — Quantitative values of indeterminate results of TB-Feron FIA, TB-Feron Elisa, and QFT-Plus. [file jcm.01486-24-s0001.docx]

**Supplementary Table 1**. Quantitative value of Indeterminate results of TB-Feron FIA, TB-Feron Elisa, and QFT-Plus

| **No** | **Methods** | **Nil TB** | **TB-Antigen** | | **Mitogen** | **TB-Antigen - Nil** | | | **Subjects** |
| --- | --- | --- | --- | --- | --- | --- | --- | --- | --- |
| 1 | TB Feron FIA | <0.145 | 0.36 | | 0.27 | 0.32 | | | Pediatrics |
| 2 |  | >10 | >10 | | >10 | -1.68 | | | Pediatrics |
| 3^#^ |  | <0.145 | <0.145 | | <0.145 | 0.13 | | | PLWD |
| 4 |  | <0.145 | <0.145 | | <0.145 | 0.09 | | | PLWD |
| 5 |  | <0.145 | <0.145 | | 0.33 | 0.07 | | | PLWD |
| 6* |  | 1.1 | 0.91 | | 1.17 | -0.19 | | | PLWH |
| 7* |  | <0.145 | <0.145 | | <0.145 | -0.04 | | | PLWH |
| 8 |  | <0.145 | <0.145 | | <0.145 | 0 | | | PLWH |
| 9 |  | <0.145 | <0.145 | | 0.32 | 0.04 | | | PLWH |
| 10 |  | <0.145 | <0.145 | | <0.145 | 0 | | | Pregnant Woman |
| 11 |  | <0.145 | <0.145 | | 0.25 | 0 | | | Pregnant Woman |
| 1^#^ | TB Feron Elisa | 0.015 | 0.018 | | 0.034 | 0.003 | | | PLWD |
| 2* |  | 0.693 | 0.674 | | 1.114 | -0.019 | | | PLWH |
| 3* |  | 0.693 | 0.663 | | 0.687 | -0.03 | | | PLWH |
|  | QFT-Plus | **Nil** | **TB 1** | **TB 2** | **Mitogen** | **TB1- Nil** | **TB2 - Nil** | **Mitogen Nil** |  |
| 1^#^ |  | 0.01 | 0.01 | 0.02 | 0.41 | 0 | 0.01 | 0.4 | PLWD |
| 2 |  | 0.08 | 0.34 | 0.4 | 0.26 | 0.26 | 0.32 | 0.18 | Pregnant Woman |

Abbreviations: *Subject has the same indeterminate result from TB-Feron FIA and TB-Feron ELISA, ^#^Subject has the same indeterminate result from TB-Feron FIA, TB-Feron ELISA and QFT-Plus, PLWH=people living with HIV; PLWD=people living with diabetes mellitus
